# MNX1 Promotes Anti-HER2 Therapy Sensitivity via Transcriptional Regulation of CD-M6PR in HER2-Positive Breast Cancer

**DOI:** 10.3390/ijms25010221

**Published:** 2023-12-22

**Authors:** Weiru Chi, Bingqiu Xiu, Min Xiong, Xuliren Wang, Pei Li, Qi Zhang, Jianjing Hou, Yuting Sang, Xujie Zhou, Ming Chen, Shuyue Zheng, Liyi Zhang, Jingyan Xue, Yayun Chi, Jiong Wu

**Affiliations:** 1 Key Laboratory of Breast Cancer in Shanghai, Department of Breast Surgery, Fudan University Shanghai Cancer Center, Shanghai 200032, Chinazhanglh0923@outlook.com (L.Z.);; 2 Department of Oncology, Shanghai Medical College, Fudan University, Shanghai 200032, China

**Keywords:** breast cancer, HER2-positive, MNX1, drug resistance, biomarker

## Abstract

Although targeted therapy for human epidermal growth factor receptor 2 (HER2)-positive breast cancer has significantly prolonged survival time and improved patients’ quality of life, drug resistance has gradually emerged. This study explored the mechanisms underlying the effect of the motor neuron and pancreatic homeobox 1 (*MNX1*) genes on drug sensitivity in HER2-positive breast cancer. From July 2017 to 2018, core needle biopsies of HER2-positive breast cancer were collected from patients who received paclitaxel, carboplatin, and trastuzumab neoadjuvant therapy at our center. Based on treatment efficacy, 81 patients were divided into pathological complete response (pCR) and non-pCR groups. High-throughput RNA sequencing results were analyzed along with the GSE181574 dataset. *MNX1* was significantly upregulated in the pCR group compared with the non-pCR group in both sequencing datasets, suggesting that *MNX1* might be correlated with drug sensitivity in HER2-positive breast cancer. Meanwhile, tissue array results revealed that high MNX1 expression corresponded to a good prognosis. In vitro functional tests showed that upregulation of *MNX1* significantly increased the sensitivity of HER2-positive breast cancer cells to lapatinib and pyrotinib. In conclusion, MNX1 may serve as a prognostic marker for patients with HER2-positive breast cancer, and its expression may facilitate clinical screening of patients sensitive to anti-HER2-targeted therapy.

## 1. Introduction

The incidence of breast cancer in women has surpassed that of lung cancer and now accounts for the highest number of cancer cases worldwide. As of 2020, approximately 2.3 million new cases of breast cancer were reported each year worldwide [1]. Among them, human epidermal growth factor receptor 2 (HER2)-positive breast cancer accounts for approximately 15–20% of all breast cancer cases. This type of breast cancer is highly aggressive and has a poor prognosis [2]. HER2 is a member of the receptor tyrosine kinase family, which includes HER1, HER2, HER3, and HER4, and they play important roles in cell proliferation, apoptosis, differentiation, invasion, and migration. HER2 can form homodimers or heterodimers with other HER family genes to activate complex cell signaling cascades, including the phosphatidylinositol 3-kinase-protein kinase B (PI3K-AKT) and RAS-MAPK pathways, to regulate cell proliferation, apoptosis, and transfer [3,4].

Trastuzumab, the first approved monoclonal antibody drug against HER2, has been the first-line standard treatment for early and metastatic HER2-positive breast cancer [5]. In addition to macromolecular monoclonal antibodies, tyrosine kinase inhibitors (TKIs) and antibody-coupled drugs (ADCs) have provided additional treatment options for HER2-positive breast cancer. Developing effective anti-HER2 therapy is one of the most important methods of improving patient prognosis. Lapatinib is a dual inhibitor of the HER1 and HER2 tyrosine kinases. Lapatinib combined with capecitabine extends the median progression-free survival (PFS) compared with capecitabine alone after trastuzumab treatment failure and disease progression (8.4 vs. 4.4 months) [6]. Pyrotinib is an irreversible TKI that targets EGFR, HER2, and HER4. PHENIX studies have shown that in patients with HER2-positive metastatic breast cancer, pyrotinib combined with capecitabine extends the median PFS by nearly 7.0 months (11.1 vs. 4.1 months, *p* = 0.001) compared with capecitabine monotherapy, thereby significantly improving the objective response rate (68.6% vs. 16.0%, *p* = 0.001) [7]. The PHOEBE trial also demonstrated that pyrotinib combined with capecitabine significantly improved PFS in patients with HER2-positive metastatic breast cancer compared with lapatinib combined with capecitabine [8]. Compared with anti-HER2 monoclonal antibody drugs, small-molecule TKIs can cross the blood–brain barrier and show excellent efficacy in patients with HER2-positive breast cancer and brain metastases.

Drugs that target HER2 have greatly improved outcomes in patients with HER2-positive tumors. However, some patients with breast cancer still develop metastatic disease after completing treatment, and some advanced patients develop drug resistance after using HER2-targeted drugs [9,10]. This drug resistance mainly includes the following mechanisms: The first mechanism is that the HER2 binding site is blocked. The p95HER2 receptor is a truncated form of the full-length p185HER2 receptor, which lacks the binding site for trastuzumab but retains kinase activity. This blocks the drug-binding region of the HER2 protein, thereby hindering effective drug–receptor binding [11]. Cells expressing p95HER2 are resistant to trastuzumab monotherapy but sensitive to TKI drugs, such as lapatinib [12]. The second mechanism involves the activation of HER2/ERBB2 gene mutations. The most common HER2 L755S mutation confers resistance to lapatinib, although the irreversible TKI neratinib is able to inhibit the proliferation of cells carrying all mutations [13]. T-DM1 and T-DXd showed durable anticancer activity in patients with HER2-mutated non-small-cell lung cancer [14]. The third mechanism is the interaction between HER2 and the estrogen receptor (ER). In HER2-positive breast cancer, HER2-targeted therapy leads to increased ER expression, which promotes cell survival [15]. The fourth mechanism is the activation of downstream pathways in the HER family. For example, when HER2 tyrosine kinase is activated, PI3K is stimulated, which leads to the activation of AKT and the mammalian target of rapamycin and subsequently results in increased cell proliferation, growth, and survival [16]. The fifth mechanism is the failure to trigger the antibody-dependent cell-mediated cytotoxicity effect. The currently reported resistance mechanisms of ADCs mainly include excessive ADC exposure stress, target antigen downregulation, antigen gene deletion or mutation, internalization pathway loss, decreased lysosomal proteolytic function, lysosomal transport protein mutation, cell cycle arrest, abnormal drug efflux transporter expression, and dysregulation [17,18]. 

Breast cancer neoadjuvant therapy is a systemic treatment for patients with early and locally advanced breast cancer before local radical treatment. The index used to evaluate the effect of neoadjuvant therapy is the pathologic complete response (pCR) rate. This index is defined as the absence of residual invasive tumor cells after treatment based on the pathology of primary breast cancer tumors and axillary lymph node surgical specimens. The general definition includes primary breast cancer with no malignant tumor cells or only in situ carcinoma components found in the lesion, whereas the strict definition also includes the absence of cancer cells in regional lymph nodes. In some subtypes, especially in patients with HER2-positive breast cancer, obtaining the pCR after neoadjuvant therapy can significantly improve the prognosis [19].

The motor neuron and pancreatic homeobox 1 *(MNX1)* gene, which is also known as *HLXB9* or *HB9*, is located on human chromosome 7 and contains three exons. The MNX1 protein has three helical regions with a structure similar to the helix-turn-helix structure. It is a key developmental gene normally expressed in neurons as well as pancreatic and lymphoid cells, and it is also involved in motor neuron differentiation and pancreatic β-cell development. Defects in this gene cause hereditary osteodystrophy. MNX1 is a transcription factor, and this gene encodes a nuclear protein that contains a homeobox domain and belongs to the HOX gene family [20,21]. Related reports on MNX1 in cancer revealed that it is involved in promoting the proliferation of squamous cervical cancer by regulating cyclin E [22]. MNX1 promotes cell proliferation in colorectal cancer by activating the Wnt/β-catenin signaling pathway and upregulating the downstream genes *c-Myc* and *CCND1* [23]. MNX1 promotes the G1-S transition of bladder cancer cells by directly binding to the promoters of *CCNE1* and *CCNE2* and upregulating their expression at the transcriptional level [24]. For breast cancer, bioinformatics analyses of MNX1 have been carried out in previous research and showed that its expression in HER2-positive breast cancer is significantly higher than that in other types of breast cancer [25]. However, these studies did not further explore the mechanism of action of MNX1. Moreover, current studies have not focused on MNX1 drug sensitivity in HER2-positive breast cancer.

Limited clinical biomarkers are available to evaluate the efficacy of neoadjuvant therapy for HER2-positive early breast cancer. Selecting a good predictive marker can not only generate more accurate prognoses but also improve the effect of therapy. The purpose of this study was to screen genes that may affect the efficacy of HER2-positive breast cancer drugs, explore their functional phenotypes in the drug sensitivity of HER2-positive breast cancer, and investigate the possible mechanisms underlying drug sensitivity. Moreover, this study aims to define the clinical significance and translational value of the investigated genes in HER2-positive breast cancer.

## 2. Results

### 2.1. Screening for Genes That May Affect Drug Efficacy in HER2-Positive Breast Cancer

**Figure 1:**
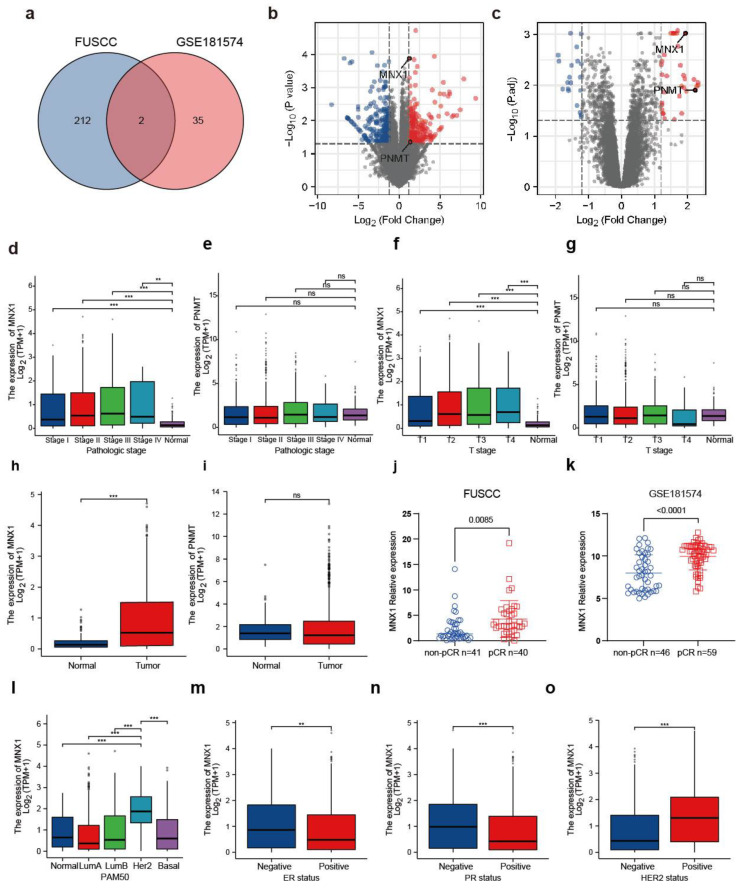
Motor neuron and pancreatic homeobox 1 (*MNX1*) serve as predictive biomarkers for neoadjuvant chemotherapy of breast cancer. (**a**) Venn diagram of upregulated genes in the pathological complete response (pCR) group of the FUSCC and GSE181574 datasets, where both phenylethanolamine N-methyltransferase (*PNMT*) and *MNX1* were included. (**b**,**c**) Volcano map of the differentially expressed genes (DEGs) (FDR < 0.05, |log2FC| > 1.2) based on the (**b**) FUSCC and (**c**) GSE181574 datasets. The locations of *PNMT* and *MNX1* are labeled with black circles. Blue circles represent downregulated genes. Red circles represent upregulated genes. (**d**,**f**) *MNX1* expression in the (**d**) pathological and (**f**) T stages measured using TCGA data. (**e**,**g**) PNMT expression in the (**e**) pathological and (**g**) T stages measured using TCGA data. (**h**) Expression of *MNX1* was significantly increased in breast cancer tissues compared with that in normal breast tissues, as measured using TCGA data. (**i**) Analysis of the relationship between PNMT in breast cancer tissues compared with that in normal breast tissues using TCGA data. (**j**,**k**) *MNX1* expression in the pCR and non-pCR groups from the (**j**) FUSCC and (**k**) GSE181574 datasets. (**l**–**o**) *MNX1* expression in (**l**) different subtypes, (**m**) estrogen receptor (ER) status, (**n**) PR status, and (**o**) HER2 status. ** *p* ≤ 0.01; *** *p* ≤ 0.001; ns—not significant.

We collected 81 core needle biopsy samples from patients with HER2-positive breast cancer before treatment. All patients received the paclitaxel or carboplatin plus trastuzumab regimen for neoadjuvant therapy at our center. Differentially expressed genes were screened using RNA high-throughput sequencing, with a *p* value < 0.05 and a |log2(fold change)| > 0.5 as the screening criteria. A total of 620 up-regulated and 645 down-regulated genes were identified. We also performed a differentially expressed gene analysis of the GSE181574 dataset, which contains 105 core needle biopsy samples before breast cancer treatment, including 36 HR−/HER2+ and 69 HR+/HER2+ cases. In this cohort, 52 cases received ado-trastuzumab emtansine plus pertuzumab (T-DM1/P), 44 received paclitaxel plus trastuzumab and pertuzumab (THP), and 9 received the paclitaxel plus trastuzumab (TH) regimen as a control arm. Differentially expressed genes in the pCR group of the two datasets were intersected on a Venn diagram with a *p* value < 0.05 and multiple fold change >1.2 to screen the drug-sensitive genes as predictors of response to neoadjuvant therapy. Data from our center and the GEO database were used to screen two genes, namely, *MNX1* and phenylethanolamine N-methyltransferase (*PNMT*) (Figure 1a–c).

Next, in the GSE52707 (in this cohort, SK-BR-3 cells were isolated using fluorescence-activated cell sorting to enrich for elevated HER2 levels and SK-BR-3 LR: SK-BR-3 cells treated with increasing lapatinib concentrations [0.2–5 μM] for several months) and GSE15043 (trastuzumab-sensitive BT474 parental cells were used as the control group, and 1.0 and 0.2 μM trastuzumab treatment was used to generate trastuzumab-resistant strains as the experimental group) datasets, it was found that the expression levels of *MNX1* and PNMT were relatively low in lapatinib- and trastuzumab-resistant cells (Figure S1a–d). This suggests that *PNMT* and *MNX1* may increase the drug sensitivity of HER2-positive breast cancer cell lines to trastuzumab and lapatinib. The *p* and fold-change values of *MNX1* were relatively high in both cohorts (GSE52707: *MNX1 p* = 0.000002, logFC = 1.2613 vs. PNMT *p* = 0.008260, logFC = 0.3779; GSE15043: *MNX1 p* = 0.000215, logFC = 0.3420 vs. PNMT *p* = 0.004340, logFC = 0.7270). We used the online ROC Plotter for ROC analysis and found that *MNX1* and *PNMT* were more highly expressed in the pCR group compared with the non-pCR group in patients receiving trastuzumab (Figure S1g). However, in lapatinib-treated patients, *MNX1* was relatively highly expressed in the pCR group compared with the non-pCR group, while PNMT expression was not markedly different between the groups (Figure S1h). TCGA database analysis revealed that *MNX1* expression was significantly higher compared with that in normal tissue in the pathological (Figure 1d) and clinical tumor stages (T stage) (Figure 1f), whereas PNMT expression was not significantly different in the pathological (Figure 1e) and T stages (Figure 1g) compared with that of normal tissue. Using the TCGA database, 772 histological types were used to draw ROC curves for invasive ductal carcinoma and 111 normal tissues. The results showed that the predictive ability of *MNX1* was more accurate (AUC = 0.721, CI = 0.684–0.758) than that of PNMT (AUC = 0.568, CI = 0.524–0.612). Using the TCGA database, 1109 tumors and 113 normal tissues were used to construct ROC curves, for which the predictive accuracy of *MNX1* was higher (AUC = 0.714, CI = 0.678–0.750) than that of PNMT (AUC = 0.543, CI = 0.501–0.586) (Figure S1f). The above results show that *MNX1* was significantly upregulated in breast cancer tissues, and the predictive performance of *MNX1* in distinguishing cancer, paracancer, invasive ductal carcinoma, and normal tissues was significantly higher than that of PNMT, suggesting that *MNX1* may be an important potential diagnostic biomarker in breast cancer. Thus, *MNX1* was selected as a candidate target for follow-up research. 

In the FUSCC and GSE181574 datasets, *MNX1* was expressed at a higher level in the pCR group compared with the non-pCR group (Figure 1j,k). The expression level of *MNX1* was used to predict the pCR rate, and it showed certain accuracy in the FUSCC (AUC = 0.693, CI = 0.5744–0.812) and GSE181574 datasets (AUC = 0.759, CI = 0.662–0.856) (Figure S1e).

Analysis of the TCGA database showed that *MNX1* was relatively highly expressed in HER2-positive breast cancer compared with other types of breast cancer (Figure 1l). According to the expression status of ER, PR, and HER2, *MNX1* was relatively highly expressed in ER-negative patients compared with ER-positive patients (Figure 1m). *MNX1* was also more highly expressed in PR-negative patients compared with PR-positive patients (Figure 1n). Moreover, *MNX1* was relatively highly expressed in HER2-positive patients compared with HER2-negative patients (Figure 1o).

### 2.2. Overexpression of MNX1 Was Correlated with Good Prognosis

**Figure 2:**
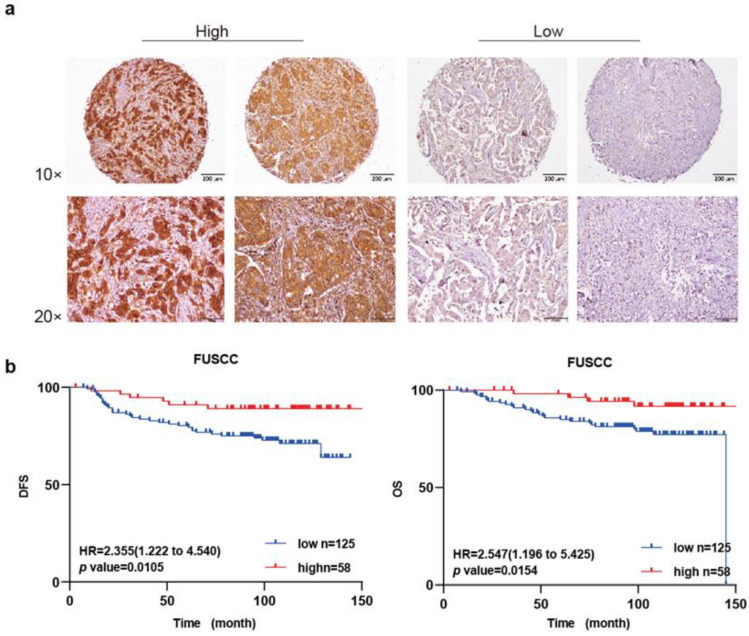
MNX1 expression level in tissue microarrays and the clinicopathologic relationship of HER2-positive breast cancer. (**a**) Immunohistochemical staining of MNX1 in tissue microarrays. From top to bottom, 10× and 20× magnifications. Scale bar: 200 µm (**b**) Kaplan–Meier 10-year disease-free (**left**) and overall survival curves (**right**) for patients with breast cancer stratified by low and high MNX1 expression.

To explore the relationship between the MNX1 protein level and clinical prognosis of patients with HER2-positive breast cancer, we used tissue microarray data of 183 patients from our center to perform immunohistochemical staining and scored the patients by the staining intensity of MNX1. Patients were then divided into MNX1 high- and low-expression groups (Figure 2a). We further plotted the survival curves of overall and disease-free survival (DFS) in patients with HER2-positive breast cancer with high or low MNX1 expression. The results showed that the overall survival and DFS of patients in the MNX1 high-expression group were significantly better than those of the low-expression group (*p* = 0.0154 and *p* = 0.0105, respectively) (Figure 2b).

The staining intensity of MNX1 in the tissue microarray and the proportion of positive cell results were combined with clinical data for analysis. The results showed that MNX1 was under-expressed in 125 tissue samples and highly expressed in 58. There was a correlation between the expression level of MNX1 and tumor size (*p* < 0.0001). In addition, the expression level of MNX1 was not related to age, menstrual status, histological grade, axillary lymph node metastasis, ER or PR status, Ki67 expression level, or surgical method (Table 1).

Next, we performed a univariate analysis on 183 patients, and the results showed that DFS was associated with axillary lymph node metastasis (*p* < 0.0001), MNX1 protein expression level (*p* = 0.013), and radiotherapy status (*p* = 0.037) (Table 2) but not associated with the patient’s age, menopausal status, histological grade, tumor size, hormone receptor level, or Ki67 level. In the multivariate analysis, we included variables with a *p* value < 0.05 and found that axillary lymph node metastasis (*p* < 0.0001) and MNX1 expression level (*p* = 0.031) were independent prognostic factors that affected HER2-positive breast cancer DFS (Table 3).

### 2.3. Overexpression of MNX1 Inhibits HER2-Positive Breast Cancer Cell Proliferation and Enhances Drug Sensitivity

**Figure 3:**
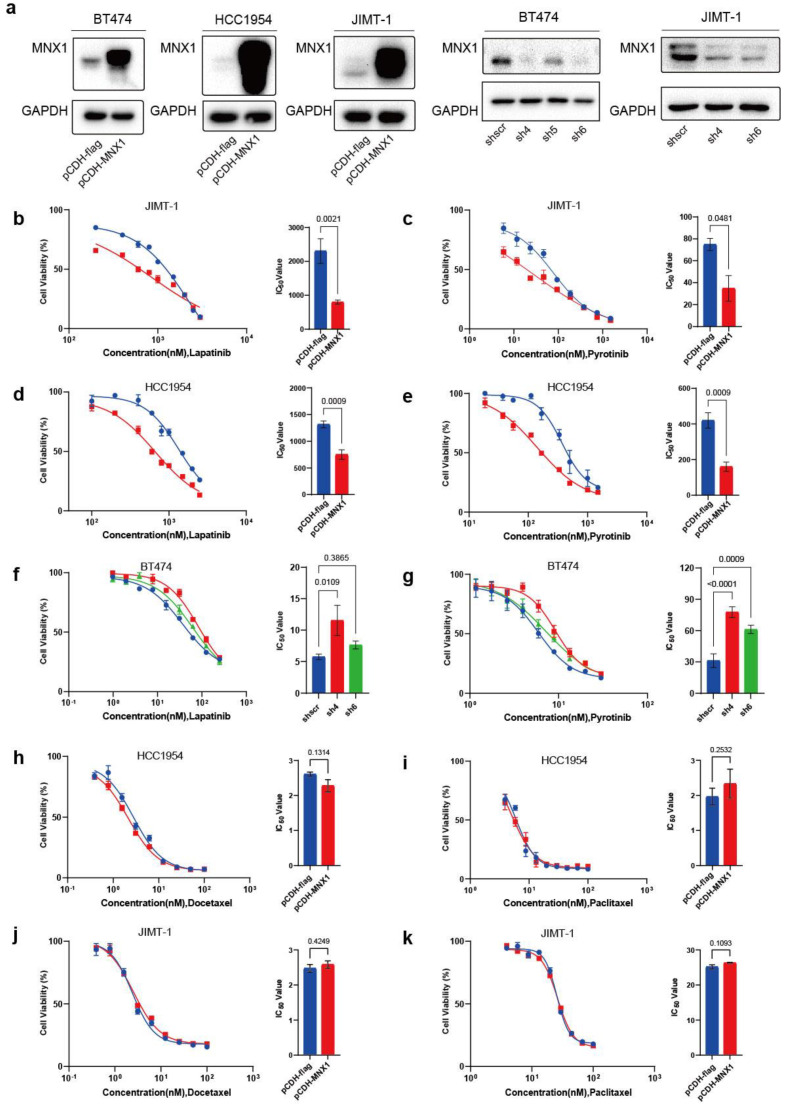
MNX1 enhances the sensitivity of HER2-positive breast cancer cells to tyrosine kinase inhibitor (TKI) drugs. (**a**) MNX1 was stably overexpressed in BT474, HCC1954, and JIMT-1 and silenced in BT474 and JIMT-1. MNX1 expression was verified using western blotting, with GAPDH as the loading control. (**b**,**c**) Half-maximal drug inhibitory concentration (IC50) of (**b**) lapatinib and (**c**) pyrotinib in stably transfected JIMT-1 MNX1 overexpression cells. (**d**,**e**) IC50 of (**d**) lapatinib and (**e**) pyrotinib in stably transfected HCC1954 MNX1 overexpression cells. (**f**,**g**) IC50 of (**f**) lapatinib and (**g**) pyrotinib in stably transfected BT474 MNX1 knockdown cells. (**h**,**i**) IC50 of (**h**) docetaxel and (**i**) paclitaxel in stably transfected HCC1954 MNX1 overexpression cells. (**j**,**k**) IC50 of (**j**) docetaxel and (**k**) paclitaxel in stably transfected JIMT-1 MNX1 overexpression cells. sh, short hairpin RNA. (**d**–**k**) The experiments were performed in triplicate, and the results are illustrated in bar graphs. (**d**–**e**,**h**–**k**) The red line represents pCDH-flag and the blue line represents pCDH-MNX1. (**f**–**g**) The blue line represents shscr, the red line represents sh4, and the green line represents sh6.

To select appropriate cell lines for the construction of stably transfected overexpression and knockdown cells, we detected *MNX1* RNA and protein expression levels in normal breast epithelial HBL100 cells and the breast cancer cell lines MDA-MB-231, T47D, BT474, SK-BR-3, HCC1954, and JIMT-1. In HER2-positive cells, MNX1 was expressed in the BT474 cell line at both the RNA and protein levels (Figure S2a,b). By detecting the expression of MNX1 in HER2-positive breast cancer cells, stably transfected MNX1 overexpression and knockdown cells were constructed from BT474 and JIMT-1 breast cancer cells. MNX1 was weakly expressed in HCC1954; therefore, only stably transfected MNX1 overexpression cells were constructed using HCC1954. Overexpression and knockdown efficiency were verified using western blotting and qRT-PCR (Figure 3a and Figure S2c). In the half-maximal drug inhibitory concentration (IC50) experiment, we found that MNX1 overexpression promoted the sensitivity of HER2-positive breast cancer cells to TKI drugs (Figure 3b,c and Figure S2d). Conversely, MNX1 knockdown resulted in reduced sensitivity to TKI drugs (Figure 3f,g and Figure S2e). Neither knockdown nor overexpression of MNX1 was sensitive to paclitaxel and docetaxel (Figure 3h–k and Figure S2h,i). The above results suggest that MNX1 can enhance the sensitivity of HER2-positive breast cancer cells to TKI drugs.

### 2.4. MNX1 Positively Regulates CD-M6PR as a Transcription Factor

**Figure 4:**
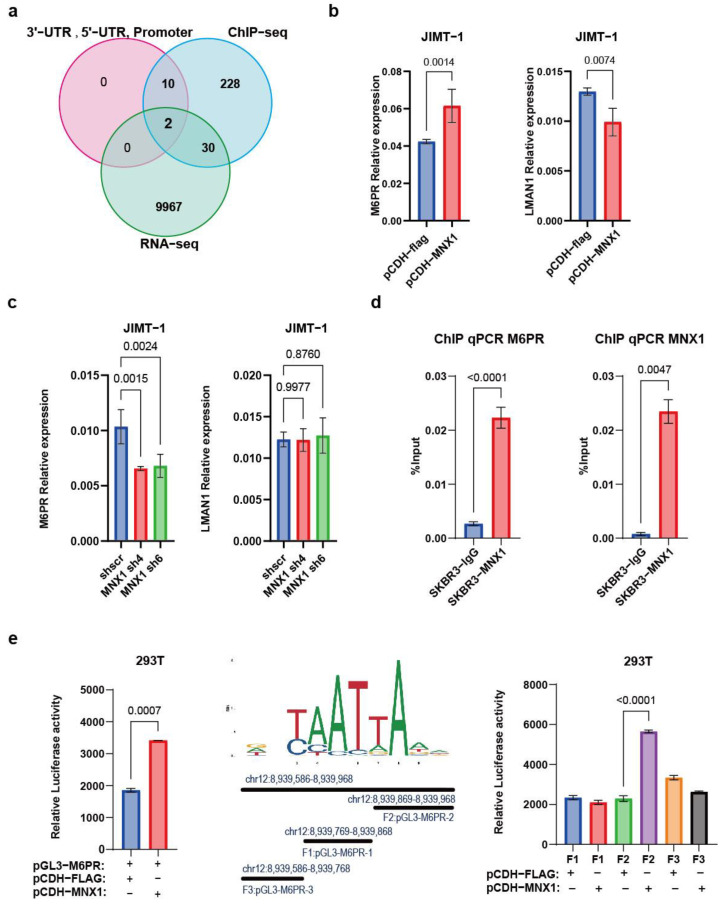
MNX1 promotes transcription of the mannose-6-phosphate receptor (M6PR). (**a**) Venn diagram of genes in the RNA-seq group, ChIP-seq group, and gene annotation at the 3′-UTR or 5′-UTR, and promoter where M6PR and LMAN1 were included. (**b**,**c**) Quantitative real-time PCR (RT-qPCR) was performed using the cDNA of stably transfected MNX1 overexpression (**b**) and knockdown cells (**c**) to detect the expression levels of M6PR and LMAN1. (**d**) Chromatin immunoprecipitation (ChIP)-qPCR was performed on ChIP samples to detect the amplification efficiency of the 3′-UTR of M6PR. (**e**) Transfection of pGL3-M6PR and pCDH-flag/pCDH-MNX1 plasmids in 293T cells for the luciferase assay (**left**). Schematic representation of fragment truncation using M6PR PEAK (**middle**) and transfection-truncated fragment and pCDH-flag/pCDH-MNX1 plasmids in 293T cells for the luciferase assay (**right**).

To further clarify the downstream target genes regulated by MNX1 as a transcription factor, we conducted chromatin immunoprecipitation sequencing (ChIP-seq) experiments and found that MNX1 binds to 12 genes in the promoter, 3′-UTR, and 5′-UTR segments. Further, combined with the RNA-seq results, it was found that two genes, *CD-M6PR* and *LMAN1*, intersected. qPCR verifications were performed on these overlapping genes, and the results showed that CD-M6PR expression increased while the LMAN1 expression level decreased as the MNX1 transcription level increased (Figure 4b). The knockdown of the MNX1 transcript level decreased CD-M6PR but did not lead to significant changes in LMAN1 (*p* = 0.876) (Figure 4c). In the following sections, CD-M6PR will be referred to as M6PR. M6PR was verified using ChIP-qPCR with ChIP samples, and the results showed that MNX1 could bind to its 3′-UTR region (Figure 4d). Following this, we explored whether MNX1 had a transcriptional activation or repression effect on M6PR. MNX1 positively regulates M6PR, as shown by dual luciferase reporter experiments (Figure 4e). To further clarify whether this was a direct or indirect transcriptional regulation, we identified binding sites for the transcription factors. We predicted possible binding sites using the JASPR database (Figure 4e) and truncated the enriched M6PR 3′-UTR into three segments to construct truncated bodies. The dual-luciferase reporter assay showed that the M6PR 3′-UTR F2 fragment may be the binding site for MNX1 (Figure 4e). 

### 2.5. Overexpression of M6PR Enhanced Drug Sensitivity of HER2-Positive Breast Cancer Cells

**Figure 5:**
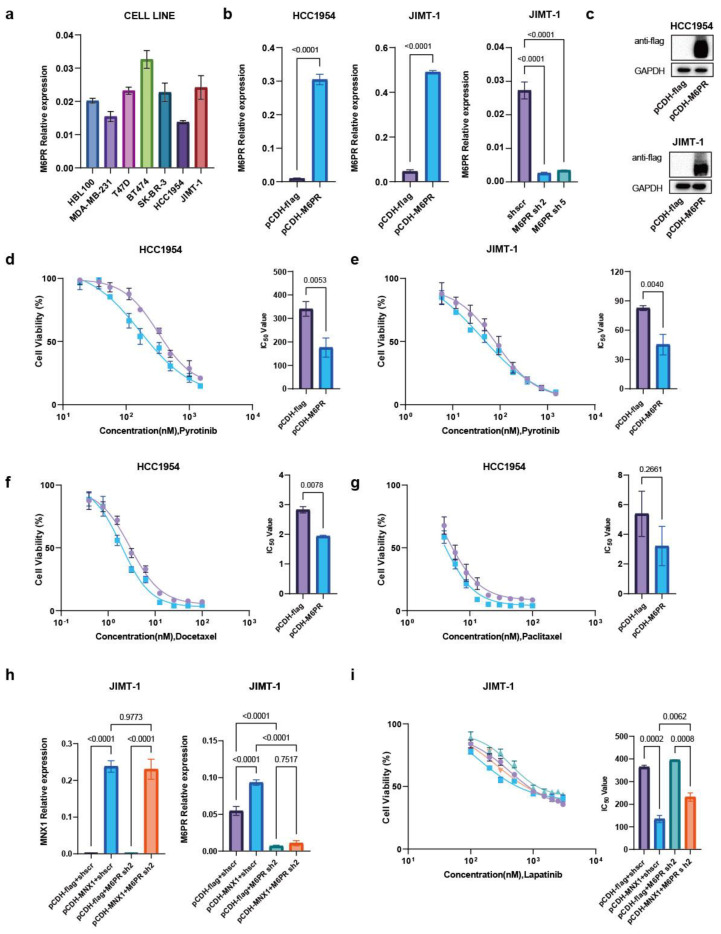
Knockdown of M6PR can reduce the sensitivity of MNX1 to lapatinib. (**a**) Bar graph showing the mRNA expression level of M6PR in breast cancer cell lines detected using quantitative real-time PCR (qRT-PCR). (**b**) Histogram showing the mRNA expression level of M6PR detected using qRT-PCR in stably transfected M6PR overexpression and knockdown cells. (**c**) M6PR was stably overexpressed in HCC1954 and JIMT-1. M6PR expression was verified using western blotting, with GAPDH as the loading control. (**d**,**e**) IC50 of (**d**) pyrotinib in stably transfected HCC1954 M6PR overexpression and (**e**) JIMT-1 M6PR overexpression cells. (**f**,**g**) IC50 of (**f**) docetaxel and (**g**) paclitaxel in stably transfected HCC1954 M6PR overexpression cells. (**h**) M6PR sh2 was transfected into JIMT-1 cells with stable MNX1 overexpression via liposome-mediated transient transfection. Histogram showing the mRNA expression level of M6PR detected using qRT-PCR. (**i**) M6PR sh2 was transfected into stably transfected JIMT-1 MNX1 overexpression cells by liposome-mediated transient transfection, and the IC50 of lapatinib was measured in stably transfected JIMT-1 MNX1 overexpression cells with M6PR sh2. (**d**–**g**,**h**) The experiments were performed in triplicate, and the results are illustrated in bar graphs. (**d**–**g**) The purple line represents pCDH-flag and the blue line represents pCDH-M6PR. (**i**) The purple line represents pCDH-flag + shscr, the blue line represents pCDH-M6PR + shscr, and the green line represents pCDH-flag + M6PR sh2. The orange line represents pCDH-MNX1 + M6PR sh2.

To select suitable cell lines to construct stably transfected, overexpressed, and knockdown cells, we detected *M6PR* RNA in normal mammary epithelial cells and breast cancer cell lines. Compared with other HER2-positive breast cancer cells, the expression of *M6PR* was similar in SK-BR-3 and JIMT-1, highest in BT474, and lowest in HCC1954 (Figure 5a). By detecting the expression of *M6PR* in HER2-positive breast cancer cells, stably transfected *M6PR* overexpression and knockdown cells were constructed in HCC1954 and JIMT-1 breast cancer cells. The overexpression and knockdown efficiency were verified using qRT-PCR and western blotting (Figure 5b,c). After overexpressing M6PR in HER2-positive breast cancer cells, the number of living cells was calculated using a CCK8 kit, and the results showed that M6PR overexpression in HCC1954 and JIMT-1 increased the sensitivity of breast cancer cells to pyrotinib (Figure 5d,e). Overexpression of M6PR was sensitive to docetaxel (Figure 5f) but did not show significant differences in sensitivity to paclitaxel (Figure 5g). M6PR sh2 was transfected into MNX1-stable JIMT-1 cells, and the efficiency of both overexpression and knockdown was verified using qRT-PCR (Figure 5h). In the rescue experiment, we found that MNX1-enhanced drug sensitivity was partially reversed by knocking down M6PR in stably transfected MNX1 overexpression cells (Figure 5i).

## 3. Discussion

Using our cohort and public databases, we screened out genes that are sensitive to the efficacy of neoadjuvant therapy in patients with breast cancer. We found that *MNX1* was highly expressed in HER2-positive breast cancer compared with other types of breast cancer. *MNX1* was highly expressed in the pCR group in both the public database and data obtained from our center, suggesting that *MNX1* may be used to predict the sensitivity of neoadjuvant chemotherapy in patients with HER2-positive breast cancer.

As previously mentioned, *MNX1* has been reported as an oncogene in multiple cancers and promotes the proliferation of cancer cells. However, it has also been reported that *MNX1* plays a dual role in childhood leukemia. For example, in infantile acute myeloid leukemia, it acts as an oncogene, whereas in childhood acute lymphoblastic leukemia, it acts as a tumor suppressor [26]. *MNX1* is a member of the HOX gene family. HOXB13 is upregulated in breast cancer [27] but exerts a cytostatic effect by negatively regulating the expression of TCF-4 in prostate cancer [28]. HOXB2 was reported to be a negative regulator of tumor growth, and its overexpression inhibited the growth of MDA-MB-231 [29]. In another study, Hur et al. found that HOXB2 was highly expressed in cancer tissues, especially in breast cancer tissues with luminal and HER2-positive molecular subtypes. The authors hypothesized that HOXB2 was dependent on molecular subtypes or could be expressed transiently to protect cells from uncontrolled proliferation in the early stages of tumorigenesis [30]. HOXB4 expression inhibits cell migration and the epithelial-mesenchymal transition (EMT) by regulating the STARD13-RhoA-ROCK signaling pathway, and its overexpression enhances the sensitivity of MDA-MB-231 and MCF7 to doxorubicin [31]. In the report by Hur et al., HOXB4 was weakly expressed in breast cancer tissues, downregulated in MCF7, MDA435, and MDA-MB-231, and highly expressed in the BT474 cell line [30]. HOXA9 is upregulated in leukemia [32] but downregulated in breast cancer, where it acts as a tumor suppressor by regulating BRCA1 expression [33]. In summary, the same HOX gene may have different functions in different cancers. In vivo tumorigenesis experiments in animals should be included in future experimental plans to observe the changes in tumor size after MNX1 overexpression and knockdown.

To explore the sensitivity of MNX1 to drug therapy, stably transfected MNX1 overexpression and knockdown cell lines were treated with various drugs at their IC50. Results showed that the MNX1-overexpressed cells were more sensitive to lapatinib and pyrotinib than the control cell line. Compared with the control cell lines, the MNX1 knockdown cell lines were less sensitive to lapatinib and pyrotinib, suggesting enhanced drug resistance. Trastuzumab and pertuzumab have different extracellular binding sites on HER2, but both act by inhibiting the formation of heterodimeric complexes between HER2 and other extracellular receptors. However, small-molecule TKI drugs, such as lapatinib, bind to the intracellular tyrosine kinase domain and directly inhibit activation of the PI3K pathway [3,34]. ADC drugs, such as T-DXd, are complexes composed of cytotoxic drugs linked to tumor-targeting monoclonal antibodies. Once released in HER2-positive breast cancer cells, the molecules diffuse out of the cells and damage surrounding HER2 tumors. The cells and tumor microenvironment produce cytotoxic effects [15,35]. To confirm that target cells are killed, in vitro experiments on the antibody-dependent cell-mediated cytotoxicity of the ADC drug-dependent microenvironment need to simulate the microenvironment or detect radioactive isotope content in solution [36]. Therefore, small-molecule TKI drugs were selected for the IC50 in vitro drug dosing experiments. Our study found that MNX1 can increase the sensitivity of HER2-positive breast cancer cells to lapatinib and pyrotinib, thereby further improving the functional phenotype of MNX1 in breast cancer. In future studies, MNX1 overexpression in animals should be investigated to observe its sensitivity to targeted drugs.

This study is the first to report that MNX1 acts as a transcription factor to activate the transcription of M6PR. M6PR plays a crucial role in transporting enzymes to lysosomes to maintain normal cellular functions. It is currently unclear whether this process has a direct relationship with the development of cancer. However, in breast cancer, the expression level of M6PR in MCF-7 was reported to be higher than that in MCF-10A and MDA-MB-231. This study found that estradiol can increase the expression of cation-dependent mannose-6-phosphate receptor (CD-MPR) and cathepsin D (CatD) and alter their distribution in MCF-7 breast cancer cells. These effects can be blocked by the anti-estrogen drug tamoxifen [37]. The functional phenotype of M6PR in cancer is also unknown. Through IC50 experiments, it was found that overexpression of M6PR increased the sensitivity of breast cancer cells to pyrotinib and docetaxel. Knockdown of M6PR in stably transfected MNX1 high-expression cells reduced the sensitivity of MNX1 to lapatinib. In summary, this study is the first to show that MNX1 enhances sensitivity to anti-HER2-targeted therapy in HER2-positive breast cancer by transcriptional activation of M6PR.

In summary, our study reveals that MNX1 enhances the sensitivity of HER2-positive breast cancer cells to anti-HER2 therapeutics. MNX1 promotes M6PR transcription by binding it to its 3′-UTR region. Therefore, in the future, it would be beneficial to evaluate MNX1 expression to screen for populations sensitive to clinical anti-HER2-targeted therapy and promote precise treatments for breast cancer.

## 4. Materials and Methods

### 4.1. Analysis of Public Data

The datasets GSE181574, GSE52707, and GSE15043 were downloaded from the Gene Expression Omnibus (GEO) database and analyzed using the GEO2R online tool. The Xiantao tool (https://www.xiantaozi.com accessed on 18 December 2023) was used to evaluate the expression of *MNX1* in breast cancer and normal tissues and in pathological and clinical tumor stages and determine the expression status of ER, PR, and HER2. Online ROC Plotter (http://rocplot.org/ accessed on 18 December 2023) was used for the ROC analysis. The Jaspar Database (https://jaspar.genereg.net/ accessed on 18 December 2023) was used to analyze the binding sites of MNX1 and CD-M6PR.

### 4.2. Patients and Tissue Samples

The research group included 81 core needle biopsy samples (CNB) that were prospectively collected from breast cancer patients who received neoadjuvant therapy at Fudan University Shanghai Cancer Center before treatment. The collection of tissue samples was reviewed by the ethics committee, and informed consent was obtained from all patients. Immediately after the core needle biopsy, the core needle puncture sample was soaked in a cryopreservation tube filled with the tissue protection solution RNAlater (Thermo Fisher Scientific, Waltham, MA, USA) and stored in a refrigerator at −40 °C until the tissue RNA was extracted. 

A total of 183 specimens from HER2-positive breast cancer patients who underwent surgery and follow-up treatment (PCH regimes) were collected at the Fudan University Shanghai Cancer Center between July 2007 and July 2012 to construct tissue microarrays (provided by the Department of Pathology). The collection of tissue samples was reviewed by the ethics committee, and informed consent was obtained from the patients. The inclusion criteria were ① female patients; ② needle biopsy-confirmed breast cancer; ③ immunohistochemically confirmed HER2 positive (immunohistochemically HER2+++ or HER2++, ++~+++, and FISH was amplified); ④ HER2-positive breast cancer-confirmed postoperative pathological specimens; and ⑤ preservation of the postoperative primary tumor wax block in the pathology department of our hospital. The tissue microarrays were wax-sealed and stored in a refrigerator at −40 °C until use.

### 4.3. Expression Plasmids, Truncated Plasmids, shRNAs, and Reagents

The pCDH-CMV-MCS-EF1-copGFP vector was digested using EcoRI-HF and BamHI-HF (New England Biolabs, Ipswich, MA, USA). pCDH-MNX1-FLAG was cloned from the BT474 cDNA using Q5 Hot Start High-Fidelity DNA Polymerase (New England Biolabs) and ligated using T4 DNA Ligase (Thermo Fisher Scientific). pCDH-M6PR-FLAG was prepared in the same manner. The pLKO.1-TRC vector was digested using EcoRI-HF and AgeI-HF (New England Biolabs). M6PR forward and reverse oligos were annealed and ligated to the pLKO.1 vector. The shRNAs against MNX1 in the pLKO.1-puro vector were purchased from GeneChem (GeneChem, Shanghai, China). Transfection of these plasmids was performed using Lipofectamine 2000 reagent (Invitrogen, Waltham, MA, USA) according to the manufacturer’s instructions. Cells (0.5 × 10^6^) were seeded and infected by a retrovirus generated by pCDH-MNX1-FLAG for 3 d. The stable cell lines were selected using 0.5 μg/mL puromycin for 7 d. Primers used are listed in the Supplementary Table S1.

### 4.4. Cell Lines and Cell Culture

BT474, JIMT-1, and HCC1954 cell lines were obtained from the American Type Culture Collection (ATCC) and characterized by Short Tandem Repeat (STR) profiling. Cells resuscitated from the original passage and passaged within 6 months were used in all experiments. The BT474 and HCC1954 cell lines were routinely maintained in Roswell Park Memorial Institute’s (RPMI) 1640 medium, and JIMT-1 was routinely maintained in Dulbecco’s modified Eagle medium (DMEM), with both containing 10% fetal bovine serum (FBS), 100 U/mL penicillin, and 100 μg/mL streptomycin. All cells were cultured at 37 °C and 5% CO_2_.

### 4.5. RNA Isolation, RT-PCR, and RT-qPCR

Total RNA was extracted from cells or tissues using TRIzol reagent (Thermo Fisher Scientific), and reverse transcription polymerase chain reaction (RT-PCR) was performed using HiScript III 1st Strand cDNA Synthesis Kit (Vazyme, Nanjing, China). Real-time quantitative polymerase chain reaction (qPCR) was performed using the SYBR qPCR Master Mix (Vazyme, China) and an ABI QuantStudio 5 system (Thermo Fisher Scientific). The relative expression of MNX1 was calculated using GAPDH using the comparative delta CT method. All reactions were performed in triplicate. Primers used in the PCR reactions are listed in the Supplementary Table S1.

### 4.6. RNA-Seq

Total RNA was collected for the RNA-Seq analysis from the cells stably expressing MNX1. For the RNA-Seq analysis of cell lines (MNX1 overexpression vs. control), raw sequencing reads were mapped against the human reference genome (Homo_sapiens.GRCh38.101). Gene expression levels were calculated based on fragments per kilobase of transcript per million mapped reads (FPKM) values.

### 4.7. Immunohistochemistry

Tissue slices were deparaffinized at 65 °C. The slices were placed in xylene and rehydrated with graded ethanol (100%–95%–75%). After using citric acid buffer (Sangon biotech, Beijing, China) for antigen retrieval, the slices were cooled to room temperature and washed with 1× PBS. Then, the slices were incubated with anti-MNX1 (Abcam, 1:500, Cambridge, UK) overnight at 4 °C. The DAB staining kit (Gene Tech, Shanghai, China) was used in the subsequent step. Images were captured using an Olympus microscope (Tokyo, Japan). The patients were stratified into high and low MNX1 expression groups based on the intensity of MNX1 tissue staining and the proportion of positive cells. Staining Intensity Score: 0 points (no staining), 1 point (weak staining), 2 points (moderate staining), and 3 points (strong staining). Percentage of Stained Cells Score: 0 points (no stained cells), 1 point (<25% of cells stained), 2 points (25–50% of cells stained), 3 points (50–75% of cells stained), and 4 points (>75% of cells stained). Composite Score: The staining intensity score and the percentage of stained cells score can be summed to obtain a composite score. The expression of MNX1 is categorized as either low expression (total score 0–3) or high expression (total score 4–7) based on the composite score.

### 4.8. Chromatin Immunoprecipitation (ChIP)

For cross-linking, cells (5 × 10^6^) were placed in a 100 mm culture dish and supplemented with medium to 20 mL. Formaldehyde (270 µL) was added, and the dish was placed on a shaker at room temperature for 10 min at a speed of 30 rpm. For termination of cross-linking, 1 mL of 1.25 M glycine was added, mixed immediately, and shaken for 5 min at room temperature. The supernatant was discarded, and the cells were washed twice with cold PBS. PBS (1 mL) containing protease inhibitors was then added, and the cells were scraped off and collected in DNA LoBind tubes (Eppendorf, Hamburg, Germany), which were centrifuged at 800× *g* for 5 min at 4 °C to collect the plaques. Cells underwent a nuclear-plasma separation experiment to remove the cytoplasm and retain the nucleus. ChIP nucleus lysis buffer (600 µL, containing protease inhibitors) was added and dispensed into 200 μL Eppendorf tubes. The cold circulating water bath was precooled to 4 °C before inserting the samples. The ultrasonic instrument was set to 30 s on and 30 s off, which was performed 10 times a cycle for a total of four cycles. The sonicated samples were combined into one tube and centrifuged at 13,000 rpm for 10 min at 4 °C. After centrifugation, 200 μL was aliquoted into new Eppendorf tubes, and 5% of the volume was reserved as the input. ChIP dilution buffer (1800 µL) and protease inhibitors (20 μL) were added to each sonicated sample (200 μL). The target antibody and control antibody IgG were added, and the samples were rotated at 4 °C for 2 h. For each reaction, 40 µL of magnetic beads were placed on a DynaMag-2 system (Thermo Fisher Scientific), the preservation solution was discarded, and the samples were washed with ChIP dilution buffer three times. A magnetic bead suspension was added evenly to the target antibody tube and the control tube and rotated for 4 h at 4 °C. The sample was placed on the DynaMag-2, the supernatant was discarded, and 1000 μL of low-salt buffer was added. The sample was then rotated at 4 °C for 5 min, and the supernatant was discarded. This process was repeated once. High-salt buffer (1000 µL) was added to wash the magnetic beads, which were rotated at 4 °C for 5 min, and the supernatant was discarded. LiCl buffer (1000 µL) was added to wash the magnetic beads, which were rotated at 4 °C for 5 min, and the supernatant was discarded. TE buffer (1000 µL) was added to wash the magnetic beads, which were rotated at 4 °C for 5 min; the supernatant was discarded; and the process was repeated once. To resolve cross-linking, the magnetic beads were placed on the DynaMag-2, and the supernatant was discarded. Then, 240 μL of ChIP elution buffer was added, and the magnetic beads were resuspended. Next, 10 μL of proteinase K was added, and the Eppendorf tube was sealed with a sealing film and kept at 55 °C overnight in a shaking dry bath. The magnetic beads were adsorbed onto the DynaMag-2, and the supernatant was transferred to a new DNA LoBind tube. For DNA purification, the Zymo Research ChIP DNA Clean & Concentrator (D5205) kit (Tustin, CA, USA) was used. First, DNA binding buffer (1250 µL) was added and mixed well. The mixture was then added to the collection column and centrifuged at 10,000× *g* for 30 s at room temperature. After discarding the waste, wash buffer (200 µL) was added to the collection column and centrifuged again at 10,000× *g* for 30 s at room temperature. This process was repeated. The collection column was placed into a new DNA LoBind tube, and 30 μL ddH20 was added. The mixture was centrifuged at 10,000× *g* at room temperature for 1 min. The collection column was then discarded, and the sample was stored at −20 °C.

### 4.9. Luciferase Activity Assay

HEK-293T cells (1 × 10^4^) were seeded in each well of a 24-well cell culture plate, and the density was approximately 70% after 12–18 h. The target plasmid (400 ng), luciferase reporter plasmid (400 ng), and Renilla plasmid (4 ng) were transfected. The Dual-Luciferase Reporter Assay System kit (Vazyme, China) was used for detection. After transfection for 24–48 h, the medium in the 24-well plate was discarded, and the plate was washed once with PBS. An amount of 100 μL 1× cell lysis buffer was added, and the cells were lysed for 5 min at room temperature on a shaker. The lysate was collected into new 1.5 mL tubes and centrifuged at 12,000× *g* for 2 min at room temperature. Luciferase substrate (100 µL) was added to each well of a 96-well white plate, and then 20 μL of lysis buffer was added to the corresponding well. A full-wavelength microplate reader was used to measure Firefly luciferase. Renilla substrate (100 µL) was added, and the Renilla luciferase was measured. The ratio was then calculated.

### 4.10. Western Blot Analysis

Cells were lysed in T-PER buffer (Thermo Fisher Scientific) with complete protease inhibitors on ice for 30 min. The protein concentration was determined using a BCA Kit (Solarbio, Beijing, China). Protein samples were loaded into a 10% sodium dodecyl sulfate polyacrylamide gel for electrophoresis (SDS-PAGE) and then transferred to 0.22 μM PVDF membranes (Merck, Darmstadt, Germany). Membranes were blocked with 5% non-fat milk and incubated with anti-MNX1 (Cell Signaling Technology, 1:1000, Danvers, MA, USA, 41983), anti-FLAG (Sigma Aldrich, St. Louis, MO, USA 1:1000, F1804), and anti-GAPDH (Proteintech, 1:10000, BOS, Rosemont, IL, USA, 60004-1-Ig). Target proteins were detected using an enhanced chemiluminescence kit (Biosharp, Anhui, China).

### 4.11. IC50 

A CCK-8 assay was used to determine the IC50. The CCK8 analysis was subsequently performed according to the standard procedure. In brief, breast cancer cells (3–8 × 10^3^ cells/100 μL) were seeded in 96-well plates. After 24 h, the cells were treated with various concentrations of DMSO solution containing lapatinib or paclitaxel, with each concentration tested in triplicate. The cells were subsequently cultured for an additional 3–7 d. After 24 h, cells were treated with pyrotinib (1500 nM, maximum concentration) or lapatinib (4000 nM, maximum concentration). The viability was measured after 3–7 d using CCK-8 for 1–4 h (Promega, Madison, WI, USA). The absorbance was measured at a wavelength of 450 nm using a microplate reader, where D represents the absorbance value. The inhibition rate of cell proliferation due to varying concentrations of the drug (y) was calculated based on the absorbance values, and the experiment was conducted in triplicate. The cell survival rate was calculated using the following formula:

Cell Survival Rate (%) = [(Experimental group D450 − Blank group D450)/(Control group D450 − Blank group D450)] × 100%

### 4.12. Statistical Analysis

The measured data are expressed as the mean ± standard deviation. The *t*-test was used to analyze the measurement data of the two groups. Univariate analysis of variance was performed for multiple measurements, and the K–W test (Kruskal–Wallis) and other statistical methods were used to analyze non-parametric data. The chi-square test was used for correlation analysis. The Kaplan–Meier method was used to draw the survival curve. Univariate and multivariate regression analyses were performed using COX regression analysis. The data were processed using IBM SPSS Statistics 26.0 software or GraphPad Prism 9 software, with *p* < 0.05 indicating significant differences.

## Figures and Tables

**Table 1:** Clinicopathological features of patients with HER2-positive breast cancer.

Factor	Cases (%)	Low (%)	High (%)	*p* Value
	*n* = 183	*n* = 125	*n* = 58	
Age				0.37
≤45	32 (17.5)	24 (13.1)	8 (4.4)	
>45	151 (82.5)	101 (55.2)	50 (27.3)	
Menstruation				0.952
Non-menopause	70 (38.3)	48 (26.2)	22 (12.0)	
Menopause	113 (61.7)	77 (42.1)	36 (19.7)	
Histological grade				0.639
G2	90 (49.2)	60 (32.8)	30 (16.4)	
G3	93 (50.8)	65 (35.5)	28 (15.3)	
ER Status				0.495
ER-negative	182 (99.5)	124 (67.8)	58 (31.7)	
ER-positive	1 (0.5)	1 (0.5)	0 (0.0)	
PR Status				0.231
PR-negative	152 (83.1)	101 (55.2)	51 (27.9)	
PR-positive	31 (16.9)	24 (13.1)	7 (3.8)	
Pathological tumor stage				<0.0001
pT1	48 (26.2)	21 (11.5)	27 (14.8)	
pT2	127 (69.4)	98 (53.6)	29 (15.8)	
pT3	8 (4.4)	6 (3.3)	2 (1.1)	
Pathological nodal stage				0.445
pN0	94 (51.4)	61 (33.3)	33 (18.0)	
pN1	40 (21.9)	26 (14.2)	14 (7.7)	
pN2	21 (11.5)	16 (8.7)	5 (2.7)	
pN3	28 (15.3)	22 (12.0)	6 (3.3)	
ki67 Status				0.299
≤20%	48 (26.2)	36 (19.7)	12 (6.6)	
>20%	77 (42.1)	48 (26.2)	29 (15.8)	
Non-detect	58 (31.7)	41 (22.4)	17 (9.3)	
Surgery type				0.856
Conservative surgery	7 (3.8)	5 (2.7)	2 (1.1)	
Mastectomy	176 (96.2)	120 (65.6)	56 (30.6)	

**Table 2:** Univariate COX regression analysis for DFS in patients with breast cancer.

Factor	HR	95% CI	*p* Value
Age			0.558
≤45 vs. >45	0.791	(0.362–1.731)	
Menstruation			0.598
Non-menopause vs. menopause	1.193	(0.619–2.296)	
Histological grade			0.282
G2 vs. G3	1.421	(0.749–2.694)	
ER Status			0.074
ER-positive vs. ER-negative	6.17	(0.836–45.556)	
PR Status			0.79
PR-positive vs. PR-negative	1.917	(0.927–3.964)	
pT			0.077
pT1 vs. pT2	0.241	(0.070–0.829)	0.024
pT1 vs. pT3	0.379	(0.132–1.090)	0.072
pN			<0.0001
pN0 vs. pN1	0.096	(0.044–0.210)	<0.0001
pN0 vs. pN2	0.099	(0.036–0.271)	<0.0001
pN0 vs. pN3	0.334	(0.132–0.848)	0.021
ki67 Status			0.469
≤20% vs. >20%	1.33	(0.614–2.880)	
Radiotherapy			0.037
Yes vs. No	1.972	(1.043–3.730)	
MNX1 expression			0.013
High vs. low	0.329	(0.137–0.787)	

HR, hazard ratio; CI, confidence interval.

**Table 3:** Multivariate COX regression analysis for DFS in patients with breast cancer.

Factor	HR	95% CI	*p* Value
pN			<0.0001
pN0 vs. pN1	0.064	(0.024–0.168)	<0.0001
pN0 vs. pN2	0.084	(0.029–0.239)	<0.0001
pN0 vs. pN3	0.37	(0.144–0.952)	0.039
Radiotherapy			0.123
Yes vs. No	0.528	(0.235–1.189)	
MNX1 expression			0.031
High vs. low	0.381	(0.159–0.917)	

HR, hazard ratio; CI, confidence interval.

## Data Availability

Additional datasets used and/or analyzed during the current study are available from the corresponding author on reasonable request.

## Supplementary Materials

The following supporting information can be downloaded at https://www.mdpi.com/article/10.3390/ijms25010221/s1.
