# Supplementary material for: MNX1 Promotes Anti-HER2 Therapy Sensitivity via Transcriptional Regulation of CD-M6PR in HER2-Positive Breast Cancer
Source: Int J Mol Sci. 2023 Dec 22;25(1):221. doi: 10.3390/ijms25010221 (PMC10778903; doi:10.3390/ijms25010221)
Supplement: Supplementary file 1 [file ijms-25-00221-s001.zip › Supplementary Figure S1.pdf]

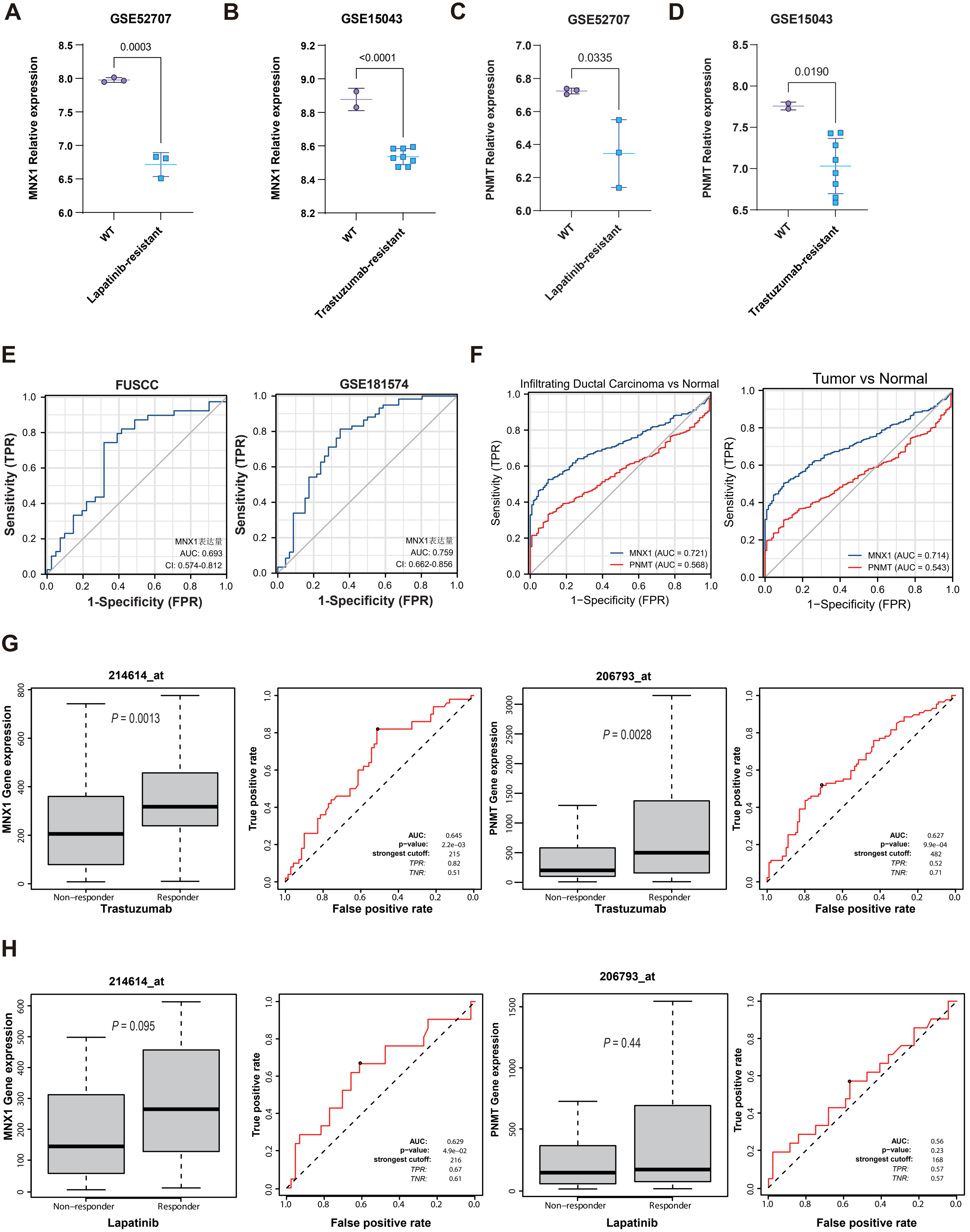

Figure S1. (A,B) MNX1 expression in the (A) lapatinib- and (B) trastuzumab-resistant strains from the GSE52707 and GSE15043 datasets, respectively. (C,D) PNMT expression in the (C) lapatinib- and (D) trastuzumab-resistant strains from the GSE52707 and GSE15043 datasets, re-spectively. (E) ROC curve analysis of MNX1 expression in predicting pathological complete re-sponse (pCR) rate in the FUSCC and GSE181574 datasets. (F) ROC curve analysis of the predictive ability of MNX1 and PNMT expression for infiltrating ductal carcinoma and para cancer, cancer and paracancer. (G,H) ROC Plotter database analyses showing the expression levels of MNX1 and PNMT in the non-pCR and pCR groups of patients receiving (G) trastuzumab and (H) lapatinib treatment. FPR, false positive rate; WT, wild-type.
