# Supplementary material for: MNX1 Promotes Anti-HER2 Therapy Sensitivity via Transcriptional Regulation of CD-M6PR in HER2-Positive Breast Cancer
Source: Int J Mol Sci. 2023 Dec 22;25(1):221. doi: 10.3390/ijms25010221 (PMC10778903; doi:10.3390/ijms25010221)
Supplement: Supplementary file 1 [file ijms-25-00221-s001.zip › Supplementary Figure S2.pdf]

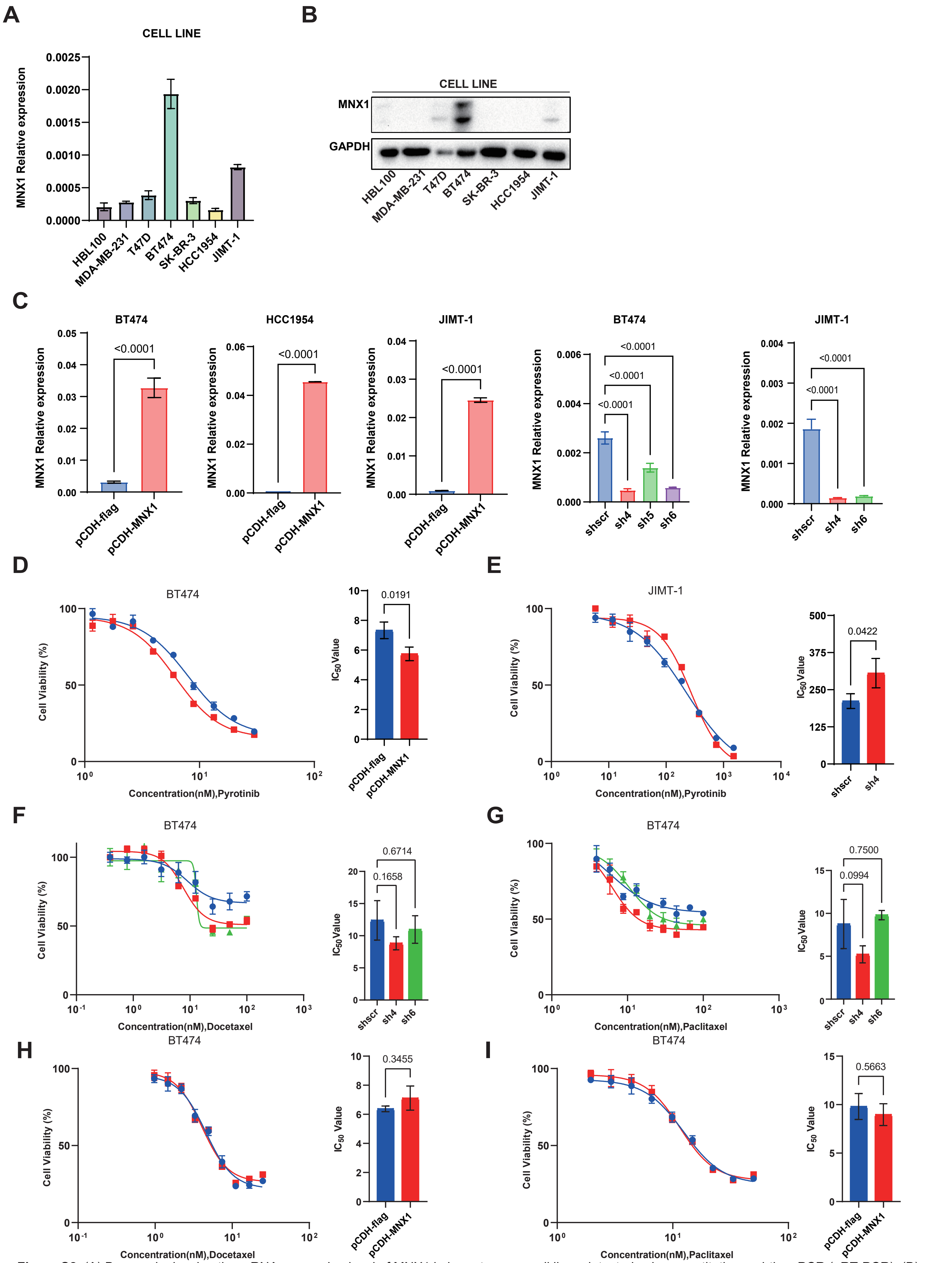

Figure S2. (A) Bar graph showing the mRNA expression level of MNX1 in breast cancer cell lines detected using quantitative real-time PCR (qRT-PCR). (B) Protein expression level of MNX1 in breast cancer cell lines detected using western blotting. (C) Histogram showing the mRNA expres-sion level of MNX1 detected using qRT-PCR in stably transfected MNX1 overexpression and knockdown cells. (D,E) IC50 values of (D) pyrotinib in stably transfected BT474 MNX1 over-expression and (E) JIMT-1 MNX1 knockdown cells. (F,G) IC50 of (F) docetaxel and (G) paclitaxel in stably transfected BT474 MNX1 knockdown cells. (H,I) IC50 of (H) docetaxel and (I) paclitaxel in stably transfected BT474 MNX1 overexpression cells.
