# Supplementary material for: MNX1 Promotes Anti-HER2 Therapy Sensitivity via Transcriptional Regulation of CD-M6PR in HER2-Positive Breast Cancer
Source: Int J Mol Sci. 2023 Dec 22;25(1):221. doi: 10.3390/ijms25010221 (PMC10778903; doi:10.3390/ijms25010221)
Supplement: Supplementary file 1 [file ijms-25-00221-s001.zip › Supplementary Table S1.pdf]

Expression Plasmids	
PCDH-MNX1-F	TTCTAGAGCTAGCgaattcatggaaaaatccaaaaatttccgcat
PCDH-MNX1-R	TGGTCTTTGTAGTCggatccctactggggcgcgggctggtggct
PCDH-M6PR-F	TTCTAGAGCTAGCgaattcatgttccctttctacagctgct
PCDH-M6PR-R	TGGTCTTTGTAGTCggatcccattggtaataaatggtcatcCCTT

shRNA for M6PR	
shM6PR-2-F	ccggCACATCTTCAACGGAAGTAATctcgagATTACTTCCGTTGAAGATGTGtttttg
shM6PR-2-R	aattcaaaaaCACATCTTCAACGGAAGTAATctcgagATTACTTCCGTTGAAGATGTG
shM6PR-5-F	ccggCCTCATCTCACCCTTACTATTctcgagAATAGTAAGGGTGAGATGAGGtttttg
shM6PR-5-R	aattcaaaaaCCTCATCTCACCCTTACTATTctcgagAATAGTAAGGGTGAGATGAGG

Primers used in the qPCR/chip qpcr reactions	
qMNX1-F	TGCCTAAGATGCCCGACTTC
qMNX1-R	AATCTTCACCTGGGTCTCGG
qGAPDH-F	AACGGGAAGCTTGTCATCAA
qGAPDH-R	TGGACTCCACGACGTACTCA
qM6PR-F	CTGGAGGACTGGACTGCTACT
qM6PR-R	CTCCTACCAAGTCGCAAGTTTT
qLMAN1-F	AGTTGAGGTGACATTTCGAGTG
qLMAN1-R	AGCTGATCCAAACACAGGGC
qchip MNX1-F	GCTACTGTGACAAGAACGAATTGAT
qchip MNX1-R	GACATCAAAGGAAGACGCTGTTAAT
qchip M6PR-F	GTGGAGCATTTAGCCCAGGT
qchip M6PR-R	AGGCAACCTGACATTCCACC
